# Time Perspectives Predict Mood States and Satisfaction with Life over and above Personality

**DOI:** 10.1007/s12144-016-9515-2

**Published:** 2016-10-19

**Authors:** Maciej Stolarski, Gerald Matthews

**Affiliations:** 1 Faculty of Psychology, University of Warsaw, Stawki Str. 5/7, 00-183 Warsaw, Poland; 2 Institute for Simulation & Training, University of Central Florida, Orlando, USA

**Keywords:** Time perspective, Mood, Satisfaction with life, Personality, Incremental validity

## Abstract

The present study aimed to test the incremental validity of Time Perspective (TP) scales in predicting satisfaction with life and mood, over and above the Big Five personality traits. It also investigated whether the new TP construct of Future Negative perspective contributed to prediction of these outcomes. Participants (*N* = 265) completed four measures: Satisfaction With Life Scale (SWLS), UWIST Mood Adjective Checklist (UMACL), a modified Zimbardo Time Perspective Inventory (ZTPI), and NEO-Five Factor Inventory (NEO-FFI). Results confirmed the incremental validity of TP, although Big Five dimensions were independently predictive of life satisfaction and certain mood scales. Past Negative TP was the strongest single predictor of life satisfaction. However, Future Negative TP was be the strongest mood predictor from the TP universe, after controlling for the Big Five and remaining TP dimensions. Findings suggest that TP is an important aspect of personality for understanding individual differences in well-being.

## Introduction

Higher subjective well-being (SWB: Diener et al. 2003) is quite strongly associated with extraversion and with emotional stability (low neuroticism). The Big Five model (McCrae 2009) lists these two factors along with conscientiousness, agreeableness and openness as the five fundamental dimensions necessary to attain a comprehensive account of personality. However, other personality traits beyond the Big Five may also play important roles in SWB. Recent work has emphasized the role of temporal perspectives in well-being. The temporal orientations that a person uses to structure their experience influence well-being (Boniwell et al. 2010; Rush and Grouzet 2012; Zimbardo and Boyd 1999). For example, Rush and Grouzet (2012) suggest that a focus on the present supports active, mindful engagement with life, and hence promotes wellbeing. The shaping of experience by past memories and by future plans may also play important roles (Zimbardo and Boyd 1999).

There are various theoretical accounts of temporal perspective, along with several different measures of the construct (Rush and Grouzet 2012; Shipp et al. 2009). The present study concerns the construct of Time Perspective (TP) developed by Zimbardo and Boyd (1999, 2008). TP describes the individual’s preferences for using past, present and future time frames to interpret important personal life events. Zhang and Howell (2011) found that TP predicted well-being over and above the Big Five, e.g., past negative perspective was associated with lower life satisfaction. We aimed to replicate and extend Zhang and Howell’s (2011) findings by examining mood as an outcome variable. Mood refers to immediate affective experience, and so contrasts with life satisfaction which is defined by longer-lasting beliefs. In the remainder of the introduction, we review the role of TP and personality in SWB, the use of mood scales as a criterion in well-being research, and the unresolved issues that the current research addresses.

### Time Perspectives, Personality and Subjective well-Being

Defined as *“the often nonconscious process whereby the continual flows of personal and social experiences are assigned to temporal categories, or time frames, that help to give order, coherence, and meaning to those events”* (Zimbardo and Boyd 1999, p. 1271), TP could be considered both as process, when conceptualized as an online cognitive framing of present experiences, and as a trait, when understood as a stable, habitual focus on particular time horizon(s), i.e., the past, the present, or the future. TP is seen as primarily social-cultural rather than temperamental in nature (Zimbardo and Boyd 1999).

Zimbardo and Boyd (1999) distinguished five TPs, measured with the Zimbardo Time Perspective Inventory (ZTPI): Past Positive, Past Negative, Present Fatalism, Present Hedonism, and Future. This model defines past TPs in terms of valence of memories, but Future has no specific valence, referring to a general orientation towards advance planning. However, thoughts of the future often concern hopes and fears, suggesting that positive and negative future dimensions might be distinguished. Carelli et al. (2011) broadened the TP universe by developing a Future Negative dimension, and slightly modifying traditional Future TP, labeling it Future Positive. They showed that a six-factor model optimized fit in a confirmatory factor analysis of their extended ZTPI.

The role of TP in well-being has increasingly drawn the attention of researchers. There is increasing evidence that individual differences in TP are linked to well-being, even with standard dimensions of personality controlled (Cunningham et al. 2015). TP predicts a variety of relevant criteria including life satisfaction (Boniwell et al. 2010; Zhang and Howell 2011), and health behaviors (Daugherty and Brase 2010). Zhang et al. (2013) showed that TP – SWB correlations were stable across multiple samples, with a total *N* of 1739. TP was predictive of multiple criteria including life satisfaction, psychological need satisfaction, self-determination, vitality, gratitude and emotion scales. High Past Positive and low Past Negative TPs were the dimensions most strongly associated with higher SWB, with high Present Hedonic, high Future and low Present Fatalistic scales showing weaker but fairly consistent associations. The association between TP and SWB is attributed to the powerful role of temporal schemas in shaping the person’s sense of self and their appraisal of significant events (Matthews and Stolarski 2015).

Given that almost all individual difference-level variables are more or less strongly loaded by major personality traits (Matthews et al. 2009), correlations between TP dimensions and SWB might be no more than a by-product of their covariance with traditional personality. Indeed, a recent meta-analysis (Kairys and Liniauskaite 2015) shows that TPs correlate with the Big Five dimensions, with particularly pronounced relationships between Future and Conscientiousness (.60), Past Negative and Neuroticism (.48), and Present Hedonism and Extraversion (.30). The Big Five also correlate consistently with various aspects of SWB. A meta-analysis showed that high Extraversion and low Neuroticism are substantially correlated (up to 0.5) with various well-being criteria (e.g., respectively, .49 and −.46 for happiness, .28 and −.38 for life satisfaction, or .44 and −.30 for positive affect), with other Big Five dimensions making smaller contributions (Steel et al. 2008). Similar roles of Extraversion and Neuroticism have been shown in studies of mood, findings that are attributed to greater sensitivity of brain reward systems in extraverts, and of punishment systems in high neuroticism individuals (Lucas and Diener 2000).

TP reflects time-oriented elements of self-concept that are not captured by the Big Five (Matthews and Stolarski 2015), and so should show incremental validity over these general personality traits in the prediction of well-being. In Zhang and Howell’s (2011) study, he TP dimensions explained an additional 13.7 % of the variance in life satisfaction beyond the Big Five traits. Past Positive, Past Negative and Present Hedonism all made significant, independent contributions to predicting life satisfaction. The Big Five Neuroticism and Extraversion dimensions also remained significant in the final equation. The incremental validity of the ZTPI over and above the Big Five traits has also been shown for health (Daugherty and Brase 2010), and for autobiographical memory for health (Ely and Mercurio 2011).

### Mood as a Criterion for Well-Being

Mood states are transient affective states that are influenced by cognitive processing of situational events (Lazarus 1999; Matthews et al. 2000). Measures of immediate mood can be distinguished from assessments of typical mood, which tend to converge with personality factors (Matthews et al. 2009). Mood response shows some stability in part because of temperamental factors (Watson 2000), but also because individuals apply cognitive schemas consistently in interpreting events. Such schemas may include the temporal frames described by TP theory (Zimbardo and Boyd 1999).

Immediate moods capture the quality of the person’s daily life experiences. By contrast, measures of typical mood may be biased by memory retrieval processes (Matthews 1992). Stolarski et al. (2014) collected longitudinal data on mood, and found that some aspects of TP were associated with biased recall; for example, individuals high in Present Hedonistic over-estimated their feelings of energy on a previous occasion. Such biases suggest a limitation of Zhang and Howell’s (2011) finding that TP predicts life satisfaction over and above personality. For example, a person high in Past Negative viewing their life circumstances through the lens of their unhappy memories might evaluate their life negatively, even though their day-to-day encounters were not particularly unpleasant.

Existing research on well-being and mood has commonly used Watson’s (2000) two-dimensional model, which distinguishes positive affect and negative affect. Life satisfaction is associated with higher typical positive affect and lower negative affect (Kuppens et al. 2008). Indeed, Jovanović (2015) suggested that typical positive and negative affect scales index affective elements of well-being directly, with life satisfaction measuring cognitive aspects. In his data, both affect scales correlated at ±.4 with life satisfaction. Using a measure of immediate mood, Ring et al. (2007) found that life satisfaction was correlated at .37 with positive affect and −.37 with negative satisfaction.

An alternate psychometric model for mood, which guided the present research, differentiates three dimensions (Matthews et al. 1990; Schimmack and Grob 2000): overall hedonic tone (pleasantness of mood) from energetic and tense arousal within a correlated-factor model. The 3-D model is useful for well-being research because it captures the experience of happiness through feeling contented, pleasant mood as well as through experiencing excited arousal and energy. Goryńska et al. (2011) conducted a meta-analysis of 23 studies (total *N* = 3733) reporting the relationships between two personality traits - Extraversion/activity and Neuroticism/anxiety – with the three mood. Both traits predicted mood: the average weighted correlations varied around the .20–.30 interval. Naturally, the magnitude of these relationships decreases when we analyze mood states in a particular mood-inducing situation (e.g., an exam; Zajenkowski et al. 2012).

### Time Perspective and Mood

Matthews and Stolarski (2015) suggest that TP could be treated as a mechanism for affective regulation. Specifically, TPs may be associated with biases in cognitive appraisal, in the use of autobiographical memory to shape current self-concept, and in the content of mental models used for anticipating future outcomes. The majority of studies of TP and well-being (e.g., Boniwell et al. 2010; Drake et al. 2008; Sailer et al. 2014; Zhang et al. 2013) have used outcome measures that refer to general, fairly stable beliefs about wellbeing and life satisfaction.

So far, only one study has analyzed relationships between TP and transient mood dimensions, using the three-dimensional model (Matthews et al. 1990). Stolarski et al. (2014) showed that TP dimensions explained between 19 and 23 % of mood variance depending on which mood dimension was considered. Past Negative TP was the strongest predictor for all three dimensions of mood, followed by Present-Hedonistic TP and (only for Energetic Arousal) Future TP. Moreover, there was some subtle interplay between specific TPs. The predicted effect of Future on energetic arousal was suppressed by the relationships of these variables with Present Hedonism, and Future moderated the relationship between Present Hedonism and Hedonic Tone). TPs also predicted biases in mood recollection and affective forecasting. These bias effects confirm that TP influences more than just the magnitude of affective response. TP systematically influences representation of emotional experience in memory, and the use of those representations to anticipate future experience.

Current research findings on TP, wellbeing and mood leave open several questions. First, it is unclear what role the overlap between the Big Five and TP might play in individual differences in mood. TP shows incremental validity in the prediction of life satisfaction with the Big Five controlled, but does TP also show incremental validity as a predictor of transient mood? Incremental validity is expected because TP is associated with a unique set of social-cognitive processes that influence well-being (Matthews and Stolarski 2015). Second, previous studies of TP and well-being have shown that the Future dimension is a significant but rather weak predictor of SWB criteria such as higher life satisfaction (Zhang and Howell 2011) with correlations commonly around .20. The small magnitude of the correlation is surprising because Zimbardo and Boyd (1999, 2008) have identified Future-oriented tendencies such as long-term planning and delay of gratification as critical for well-being over the life span. Carelli et al.’s (2011) model of TP, including two future-oriented scales representing positive and negative perspectives, may do a better job of assessing those elements of future TP which contribute to well-being, including higher life satisfaction and positive mood. Third, Stolarski et al.’s (2014) findings suggested another possible explanation for the limited impact of Future on mood, i.e., that there is an interplay between Future and Present Hedonic TPs associated with the moderator and interaction effects described in the previous paragraph. These somewhat complex findings require replication to determine whether they are significant for understanding the role of TP in well-being.

### Hypotheses

Based on prior research and theoretical considerations mentioned above, we hypothesized that (H1) TP dimensions would predict both life satisfaction and each of the three mood dimensions as in previous studies (e.g., Stolarski et al. 2014; Zhang and Howell 2011). We expected that Past Negative would be the most predictive single TP of these criteria, correlating with poorer mood and lower satisfaction. We expected smaller-magnitude associations between Past Positive TP, better mood and higher satisfaction, with more limited correlations between other TPs and the well-being criteria. We hypothesized (H2) that inclusion of Carelli et al.’s (2011) Future Positive and Negative dimensions would increase the power of TP to predict life satisfaction and mood. Furthermore, we aimed to determine whether (H3) the effect of TP on life satisfaction and mood remains significant after controlling for the Big Five personality dimensions. Finally we attempted to replicate the additional effects obtained by Stolarski et al. (2014), i.e., (H4) the suppression effect of Present Hedonism on Future for Energetic Arousal and (H5) the interaction between Present Hedonism and Future for Hedonic Tone.

## Method

### Participants

The sample comprised 265 Caucasian adults of Polish nationality, mainly students and their families. 143 of them were females, aged between 18 and 47 (*M* = 22.55, *SD* = 3.38) and 122 were males, aged between 18 and 51 (*M* = 22.75, *SD* = 3.95). Participants were recruited by a team of four specially trained pollsters, students of a M.A. program in Psychology at University of Warsaw, hired to support a research grant conducted by the corresponding author of the present article. They were asking potential participants individually, actively inviting them to take part in the study. After test completion participants were asked to recommend one or two potential subjects to take part in this research. The sample size and composition was very similar to that reported by Stolarski et al. (2014). Participation was voluntary; subjects were not rewarded.

### Measures

*Mood* was measured using the UWIST Mood Adjective Check List (UMACL, Matthews et al. 1990), in the Polish adaptation by Goryńska (2005). The scale has three subscales measuring: Energetic Arousal (EA), Tense Arousal (TA), and Hedonic Tone (HT). Respondents rated the degree to which each of the adjectives described their current mood on a four-point Likert-type scale. The Polish version showed adequate internal consistency (alphas ranging from .79 to .92) and sufficient convergent validity (e.g., associations with Positive/Negative Affect, state anxiety and the Big Five). Convergent validity is similar to the original, English-language scale (Goryńska 2005).

*Satisfaction With Life* was assessed using Satisfaction With Life Scale (SWLS; Diener et al. 1985) in the Polish adaptation by Juczyński (1999). It consists of five items scored with a 7-point Likert-type response format measuring global cognitive judgments of satisfaction with one’s life. Evidence on its validity is presented by Juczyński (2001), and includes correlations with other well-established indicators of well-being and psychological health.

#### Time Perspective

Zimbardo and Boyd’s ZTPI (Zimbardo and Boyd 1999), in the Polish adaptation by Kozak and Mażewski (2007), assessed traits for TP. It has 5 scales: Past Negative (PN), Present Hedonistic (PH), Future (F), Past Positive (PP), and Present Fatalistic (PF). Respondents rate their degree of endorsement of each statement on a five-point Likert scale. Additionally, we used Future Negative (FN) items provided by Carelli et al. (2011), and, consequently, we reduced the F scale by two items, which are included in FN, leaving what Carelli labels Future Positive (FP) scale. All items were translated and back-translated by certified translators. Convergent validity studies revealed a pattern of associations with other self-report scales similar to the one obtained for the original version. The six-factor solution, applied in the present study, provided better fit-to-data than the classic five-factor solution (see Jochemczyk et al. 2016).

#### Personality

The Big Five personality traits (Neuroticism, Extraversion, Openness to experience, Agreeableness and Conscientiousness) were measured with the NEO-FFI questionnaire (Costa and McCrae 1992) in the Polish adaptation by Zawadzki et al. (1998). The Polish version’s validity was demonstrated through convergence with well-established personality measures as well as high agreement between self-reports and observer ratings. Internal consistencies of the scales ranged between .68 and .86 and were comparable with those reported for the original version.

### Procedure

Participants were informed that they were to take part in a study of emotions and time orientation. Questionnaires were administered either on a group basis, in a university class setting, or individually, in home settings. UMACL was administrated first, followed by ZTPI and NEO-FFI; the order of the latter two measures was counterbalanced, in order to control for any order effects. Mood was measured first in order to avoid any effect of completing the remaining questionnaires on the state mood measure.

## Results

Descriptive statistics and Cronbach alphas for the measures and zero-order correlations are provided in Table 1.

**Table 1:**
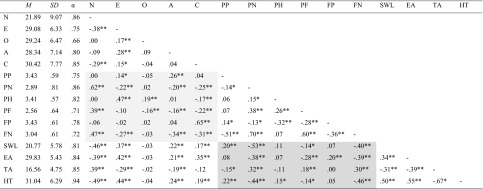
Descriptive statistics and intercorrelations

N – Neuroticism, E – Extraversion, O – Openness, A – Agreeableness, C – conscientiousness, PP – Past-Positive, PN – Past-Negative, PH – Present-Hedonistic, PF – Present-Fatalistic, FP – Future-Positive, FN – Future-Negative, EA – Energetic Arousal, TA – Tense Arousal, HT – Hedonic Tone, SWL – Satisfaction With Life. Big Five – TP relationships are shadowed in light grey, TP – mood/SWL relationships are shadowed in dark grey

* p < .05, ** p < .01

Major correlations between the Big Five and TP included those between Neuroticism and Past Negative (.62), Extraversion and Present Hedonistic (.47) and Future Positive and Conscientiousness (.65). The original ZTPI Future dimension, correlated .98, *p* < .001, with Future Positive, suggesting the two scales index the same latent variable. A novel finding was that Future Negative correlated with four Big Five dimensions, with a particularly strong relationship with Neuroticism. When we regressed Future Negative onto the Big Five, only Neuroticism proved a significant predictor, β = .64, *p* < .001. The high N-loading of Future Negative reflects the fact that anxiety, which remains one of the main facets of Neuroticism, is a “future-oriented” emotion (Zaleski 1996). Table 1 also shows that Past Negative was the TP dimension most strongly associated with (lower) SWLS scores. Associations of smaller magnitude were also found for the new Future Negative dimension, Past Positive and Present Fatalistic.

Table 1 shows that the SWLS, the Big Five and TP were all predictive of mood. Higher SWLS scores were associated with general elevation of mood, with the strongest correlation found with the UMACL Hedonic Tone scale. Big Five data were also consistent with expectation. Extraversion and low neuroticism were associated with better mood on all three UMACL scales, as in the Goryńska et al. (2011) meta-analysis. Conscientiousness was more selectively associated with higher Energy and Hedonic Tone, but not with Tense Arousal. As in Stolarski et al.’s (2014) study, at the zero-order correlations level, Past Negative was the most distinct correlate of mood, followed by Present Fatalistic and Past Positive. The relationships for Present Hedonism were weaker, and the effect of Future on Energy, which was suppressed in the earlier study, this time turned out significant already in zero-order correlations. Future Negative was also substantially correlated with poorer moods.

Further, we conducted a stepwise regression analyses to reveal the criterion validity of TP dimensions for each mood dimension and SWLS score, as well as TP’s incremental validity over and above the Big Five (see Table 2). As some of the introduced predictors were substantially intercorrelated, we checked for multicollinearity issues. However, no evidence for multicollinearity was found (all the VIF values were <2.0, and therefore were well below the critical value of 10.0, suggested by Field (2009)). In each analysis we present two alternative sets of variables introduced in step 1, showing standardized β coefficients for the Big Five and TP, respectively. In the second step we report the models including the remaining variable set: TP, if the Big Five was entered in the first step, and Big Five, if TP was entered first. The rightmost column of the table shows the full model statistics, as well as step 2 change statistics, for both orders of entry.

**Table 2:** Regression models predicting SWL and mood dimensions with Big Five traits and TPs

	β	*p*		β	*p*	
Model 1. Dependent variable: Satisfaction With Life						
*Step 1*				*Step 2*		
N	-.36	.001	*F*_(5259)_ = 19.358	-.16	.036	*F*_(11,253)_ = 14.139
E	.19	<.001	p < .001	.14	.038	p < .001
O	-.07	.186	*R* ^*2*^ = .27	-.06	.286	*R* ^*2*^ = .38
A	.15	.006		.09	.091	TP over B5:
C	.02	.692		-.03	.660	*F*_*change*(6253)_ = 7.398
PP	.11	.033	*F*_(6258)_ = 21.934	.08	.147	p < .001
PN	-.46	<.001	p < .001	-.38	<.001	*ΔR* ^*2*^ = .11
PH	.16	.004	*R* ^*2*^ = .34	.10	.131	B5 over TP:
PF	.05	.360		.09	.144	*F*_*change*(5253)_ = 3.506
FP	.06	.261		.07	.314	p = .004
FN	-.14	.039		-.04	.592	*ΔR* ^*2*^ = .04
Model 2. Dependent variable: Energetic Arousal						
*Step 1*				*Step 2*		
N	-.20	.001	*F*_(5259)_ = 22.931	.01	.887	*F*_(11,253)_ = 12.613
E	.29	<.001	p < .001	.28	.000	p < .001
O	-.08	.157	*R* ^*2*^ = .30	-.10	.057	*R* ^*2*^ = .35
A	.11	.050		.08	.163	TP over B5:
C	.24	<.001		.17	.017	*F*_*change*(6253)_ = 3.090
PP	.03	.558	*F*_(6258)_ = 15.031	-.01	.874	p = .006
PN	-.18	.016	p < .001	-.12	.100	*ΔR* ^*2*^ = .05
PH	.17	.004	*R* ^*2*^ = .26	.04	.522	B5 over TP:
PF	-.11	.077		-.11	.082	*F*_*change*(5253)_ = 7.455
FP	.20	.001		.07	.380	p < .001
FN	-.26	<.001		-.17	.029	*ΔR* ^*2*^ = .09
Model 3. Dependent variable: Tense Arousal						
*Step 1*				*Step 2*		
N	.33	<.001	*F*_(5259)_ = 12.164	.14	.090	*F*_(11,253)_ = 7.812
E	-.13	.038	p < .001	-.06	.401	p < .001
O	.02	.758	*R* ^*2*^ = .19	.02	.751	*R* ^*2*^ = .25
A	-.12	.036		-.10	.086	TP over B5:
C	.00	.954		.00	.994	*F*_*change*(6253)_ = 3.580
PP	-.15	.009	*F*_(6258)_ = 12.691	-.12	.040	p = .002
PN	.07	.371	p < .001	.00	.967	*ΔR* ^*2*^ = .06
PH	-.09	.132	*R* ^*2*^ = .23	-.05	.469	B5 over TP:
PF	.05	.398		.02	.790	*F*_*change*(5253)_ = 1.740
FP	.01	.872		.01	.886	p = .126
FN	.38	<.001		.31	.000	*ΔR* ^*2*^ = .03
Model 4. Dependent variable: Hedonic Tone						
*Step 1*				*Step 2*		
N	-.36	<.001	*F*_(5259)_ = 26.400	-.21	.006	*F*_(11,253)_ = 15.230
E	.28	<.001	p < .001	.21	.001	p < .001
O	-.10	.065	*R* ^*2*^ = .34	-.08	.129	*R* ^*2*^ = .40
A	.13	.012		.10	.074	TP over B5:
C	.04	.473		.01	.883	*F*_*change*(6253)_ = 4.260
PP	.17	.002	*F*_(6258)_ = 19.988	.13	.016	p < .001
PN	-.26	<.001	p < .001	-.16	.026	*ΔR* ^*2*^ = .06
PH	.17	.003	*R* ^*2*^ = .32	.07	.306	B5 over TP:
PF	.03	.573		.08	.207	*F*_*change*(5253)_ = 6.817
FP	.06	.270		.04	.553	p < .001
FN	-.31	<.001		-.17	.025	*ΔR* ^*2*^ = .08

N – Neuroticism, E – Extraversion, O – Openness, A – Agreeableness, C – conscientiousness, PP – Past Positive, PN – Past Negative, PH – Present Hedonistic, PF – Present Fatalistic, FP – Future Positive, FN – Future Negative, EA – Energetic Arousal, TA – Tense Arousal, HT – Hedonic Tone, B5 – Big Five

At step 1, the TP scales explained 34 % of the variance in SWLS, with several dimensions making significant contributions to the equation. Inclusion of the Big Five at Step 2 added an extra 4 % to the variance explained. In the final equation, Past Negative was the only TP dimension with a significant *β* value; Neuroticism and Extraversion made smaller but significant independent contributions. With the ordering of the steps reversed, the Big Five explained 27 % of the variance, and TP an additional 11 %, confirming that TP has incremental validity in predicting life satisfaction, over the Big Five, supporting H1. The TP scales predicted each of the three mood dimensions, explaining 23 % of variance in Tense Arousal, 26 % in Energetic Arousal, and 34 % in Hedonic Tone. H1 was thus confirmed. Out of the TP variables, Future Negative had the highest *β* value for each mood criterion, with or without controlling for Big Five. Thus, H2 was fully confirmed. Mood correlates of the remaining TP scales were similar to those reported by Stolarski et al. (2014), with the exception of Past Positive, which was a significant predictor of both Tension and Hedonic Tone here.

H3 was also confirmed, with TP predicting 5–6 % of variance in each mood dimension over and above the Big Five. With the Big Five controlled, Future Negative was the only significant predictor of all three mood scales. Past Positive was uniquely associated with lower Tense Arousal and higher Hedonic Tone, and Past Negative with the latter only. The Big Five predicted Energetic Arousal and Hedonic Tone above TP (9 % and 8 % of incremental variance, respectively), but not Tense Arousal, suggesting that TP is probably more important for this negative mood indicator than personality.

**Fig. 1:**
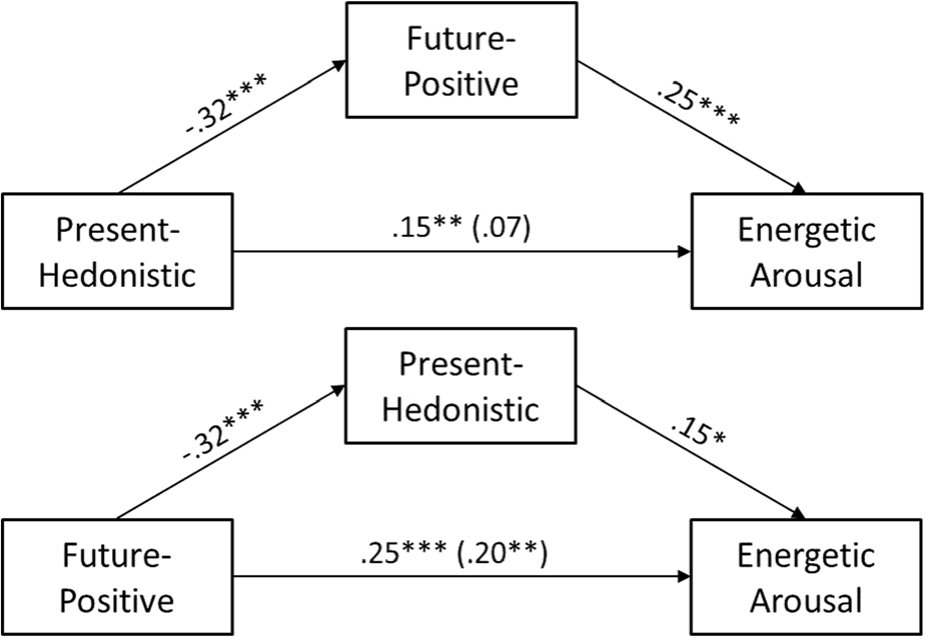
Reciprocal suppression effect of Present Hedonism and Future TPs in predicting Energetic Arousal.

To test H4 we checked for the suppression effect of Present Hedonism on Future (Positive) in predicting Energy (see Fig. 1), reported by Stolarski et al. (2014). The .20, *p* < .01 effect of Future Positive on Energetic Arousal (*c* path) changed to .25, *p* < .001 after controlling for Present Hedonistic TP (*c’* path). We tested the significance of this suppression using bootstrapping procedures. Unstandardized indirect effects were computed for each of 10,000 bootstrapped samples, and the 95 % confidence interval was computed by determining the indirect effects at the 2.5th and 97.5th percentiles. The bootstrapped unstandardized indirect effect was −.049, and the 95 % confidence interval ranged from −.124 to −.004. Thus, the suppression effect was statistically significant. Given that this time the total effect of Present Hedonism on Energetic Arousal was not significant (see Table 1), we also conducted a complementary analysis, treating Future Positive as a potential suppressor of the relationship between Present Hedonism and Energy. Again, we found an evidence for suppression effect; the non-significant .07 effect of Present Hedonism on energy (*c* path) increased to .15, *p* = .01 (*c’* path). The bootstrapped unstandardized indirect effect was −.079, and the 95 % confidence interval ranged from −.144 to −.036. Thus, the suppression effect was again statistically significant. There is seemingly a reciprocal suppression effect here, where Future Positive and Present Hedonistic suppress each other in predicting Energetic Arousal. The H4 hypothesis was confirmed. However, this suppression was not present after controlling for personality. Also, we found no evidence for any interaction of Future Positive and Present Hedonistic TPs in predicting Hedonic Tone (or any other mood dimension), using the same regression method as Stolarski et al. (2014). Therefore, H5 was not supported.

## Discussion

The present study of dispositional predictors of well-being replicated and extended existing results. Multiple TP dimensions were associated with the well-being criteria, with Past Negative TP showing the highest though negative bivariate correlations, supporting H1. The new Future Negative TP dimension (Carelli et al. 2011) contributed to prediction of well-being, especially for mood criteria, supporting H2. We replicated Zhang and Howell’s (2011) finding that TP predicts life satisfaction over and above the Big Five. The ZTPI also had incremental validity in predicting mood states, as hypothesized (H3). However, we also identified differing sets of predictors for life satisfaction and for the mood scales, suggesting that the criterion measures are to some extent picking up different aspects of wellbeing.

We confirmed (H4) a reciprocal suppression effect on the influence of TP on Energetic Arousal. Future Positive and Present Hedonistic both predict Energetic Arousal, but these two TPs are negatively correlated, producing suppression of their bivariate associations with energy. However, we did not replicate Stolarski et al.’s (2014) finding of an interactive effect of these two TPs, contrary to H5.

### Dispositional Factors in Well-Being

Findings with life satisfaction as a criterion broadly replicated previous findings, including the well-established roles of extraversion and neuroticism (Steel et al. 2008). Consistent with the Steel et al. meta-analysis, we also found smaller significant positive correlations between life satisfaction and Conscientiousness and Agreeableness; however, only the Agreeableness association remained significant in the regression analysis (with Big Five entered at Step 1). Relationships between the Big Five and TP were very similar in direction and magnitude to those found in Kairys and Liniauskaite’s (2015) meta-analysis, demonstrating the need to test for incremental validity of TP.

The pattern of correlations between TPs and the SWLS was similar to the one reported by Zhang and Howell (2011). Consistent with H1, greater life satisfaction was associated with lower Past Negative, and higher Past Positive. However, the coefficients differed in magnitude to the earlier study. In Zhang and Howell’s data correlations between SWLS scores and Past Negative (−.44) and Past Positive (−.41) were of similar magnitude; here, Past Negative was the more predictive TP dimension. We also found a substantial negative correlation between SWLS and the new dimension of Future Negative: Zhang and Howell (2011) found a weaker but significant correlation of .15 between the original Future dimension and SWLS. We replicated Zhang and Howell’s (2011) finding of incremental validity; in their study, TP added 14 % to the variance explained to the Big Five, similar to the present data, Past Negative was the only TP to remain significant in the final equation. We could not replicate independent contributions of Past Positive and Present Hedonism to well-being, although their study (*N* = 754) had greater statistical power.

These findings support the view that life satisfaction is more than just a reflection of basic temperaments. Satisfaction is also influenced by the temporal framing of experience, and the self-regulative strategies that follow from the individual’s orientation in time (Matthews and Stolarski 2015). The role of Past Negative is especially salient, and may reflect the memory retrieval processes through which the current self-concept (or ‘working self’) draws on autobiographical memory (Conway and Pleydell-Pearce 2000). For example, a child habitually criticized by his or her parents might construe their present status as an adult as failing to live up to parental expectations, despite objective accomplishments.

The study also supported Carelli et al.’s (2011) division of future TP into separate positive and negative scales, consistent with each temporal orientation having positive and negative aspects (Rush and Grouzet 2012). Positive and negative future TPs were only modestly correlated (.36), suggesting that future orientations are differentiated by affect just as past TPs are. Interestingly, the intercorrelation of past and future TPs was much stronger for the two negative TPs (.70), than for the two positive orientations (.14). Dwelling on the troubles of the past appears to project forward into the future more strongly than a focus on positive memories. The bivariate SWLS data suggested that Future Negative TP may depress satisfaction, but this TP did not make a significant contribution to prediction with the Big Five controlled.

### TPs as Predictors of Mood State

The mood data show some similarities and some differences to the life satisfaction findings. The bivariate correlations suggest that TPs that predict life satisfaction also predict transient mood states; correlation magnitudes tended to be largest for Past Negative and Future Negative. Again, TP had incremental validity over the Big Five in predicting all three mood dimensions, but some differences in detail were apparent. For SWLS, TP appeared to have a larger incremental effect than the Big Five (11 % vs. 4 %). For mood, the incremental effects of these two types of construct were more similar in size. One explanation is that life satisfaction is intended to be a primarily cognitive-judgmental rather than an affective construct (Diener et al. 1985), whereas moods are affective in nature. The Big Five may be relatively more reflective of temperamental affect, whereas TP may be more infused by cognitive self-regulatory processes (Matthews and Stolarski 2015). Thus, predictive power of these constructs may reflect the balance of affective and cognitive elements of criterion variables.

In Stolarski et al.’s (2014) study Past Negative was the strongest predictor of moods. Here, both Past Negative and Future Negative were the TP scales most strongly correlated with mood. However, the regression analyses showed that the Future scale was more consistently predictive than the Past scale, although the two scales were substantially correlated. A possible explanation is that moods are future-oriented to the extent that they represent adaptation to immediate challenges. For example, energy prepares the person for vigorous, goal-directed action, and tension represents preparedness for threat (Thayer 1997). The Future Negative scale items (Carelli et al. 2011) focus on anticipated failures to plan effectively: perceived lack of preparedness may powerfully influence mood.

The mood data also differed from the SWLS data in that TPs additional to Past Negative were independently predictive of criteria in the regression analyses. Predictors varied across the three mood dimensions. First, for Energetic Arousal, the regression model (TP only) suggested that Present Hedonism and Future Positive are energizing whereas Past Negative and Future Negative orientations drain energy. The temporal bases of energy appear complex. After controlling for the Big Five, only Future Negative made a significant contribution. The Future Negative scale items (Carelli et al. 2011) focus on anticipated failures to plan effectively. Perceived lack of preparedness for impending threats will lead to decreased levels of motivation and energy. On the other hand, although Future Positive and Present Hedonism were significantly related to increased energy, and we even replicated the suppression effect reported by Stolarski et al. (2014), their associations with this mood dimension may be ascribed to their covariance with personality traits (Extraversion and Conscientiousness in particular). The negative association between Future Positive and Present Hedonism confirms the view that living for the moment and planning for the future tend to be conflicting orientations (Zimbardo and Boyd 2008).

There were two significant TP predictors of Tense Arousal: Past Positive and Future Negative, both remaining significant after controlling for the Big Five. Stolarski et al. (2011) proposed that Past Positive is an effective and ‘emotionally intelligent’ mood regulation strategy: retrieving happy memories is one of the most effective ways to regulate bad moods (Josephson 1996). The second significant predictor – Future Negative – seems to represent a fundamental condition for increased feelings of tension, as expected from the nature of the mood dimension. Feelings of being stressed or anxious are usually induced by events or stimuli that are expected to happen in one’s future; Thayer (1997) identified tension with preparedness for threat. Indeed, Aristotle (350 B.C. / Aristotle 1954) claimed that *“Fear may be defined as a pain or disturbance due to a mental picture of some destructive or painful evil in the future”*.

Finally, four TPs predicted the intensity of Hedonic Tone. Past Negative and Future Negative were detrimental to momentary hedonic experience, whereas Past Positive and Present Hedonism were positively correlated with this mood dimension. After controlling for the Big Five, only the latter effect was not significant. This pattern of results is similar to the one obtained by Zhang and Howell (2011) for life satisfaction. The positive effect of Past Positive is probably based on the same mechanism as in the case of energy, i.e., this TP may act as a strategy for emotional regulation (Matthews and Stolarski 2015). The detrimental effects of both future dimensions show that pessimistic subjective views of one’s past and future result in decreased mood in the present.

### Limitations and Future Directions

Naturally, our study has all the limitations particular to self-report measurement and cross-sectional design. Associations between personality and SWB may be inflated by shared evaluative biases (Schimmack et al. 2008). The design allows only for a snapshot of mood on a single occasion. Future research might distinguish trait and state elements of TP in everyday contexts, e.g., through using a longitudinal experience sampling method (Rush and Grouzet 2012). Future studies should seek for potential mediators of the obtained relationships, i.e., specific mechanisms and processes that may be sensitive to the individual’s TP, focusing especially on retrieval of autobiographical memories and on mood-regulation strategies congruent with the TP (Matthews and Stolarski 2015). Research illustrating how the obtained relationships change depending on situation (e.g., whether Present Hedonistic would be more predictive for mood during a party, and Future Positive during an exam) could be also very interesting. Finally, the sample comprised mainly students and their families which naturally limits generalizability of the present results. Although such situation is common in research on TP (e.g., Zimbardo and Boyd 1999; Stolarski 2016), it limits generalizability of the present findings, thus it would be desirable to replicate the present study on a big, representative sample or on a few differentiated samples.

Authors aiming to replicate the present results should take into account the fact that mood states are sensitive to various contextual factors as established in other UMACL research with Polish student samples (e.g., Goryńska et al. 2015). While collecting data, they should be careful to create a neutral testing session without salient reinforcers and to control for contextual influences. The problem refers particularly to studies conducted online. The main disadvantage for the online methodology is that there is no way to establish the control of situational factors necessary for mood assessment, as opposed to say, stable personality traits. We do not know what participants are doing prior to (or even during) the mood assessment, and there is no way to check compliance with any instructions regarding the assessment context.

## Conclusions

The present study illustrates the incremental effect of TP dimensions in predicting well-being and over and above personality. The way in which people perceive their past, present and future remains significant for both cognitive and affective aspects of well-being, consistent with the proposal that TP is a mechanism for self-regulation (Matthews and Stolarski 2015). Past Negative has the major influence on life satisfaction, whereas additional TPs, including Future Negative, contribute to predicting temporary mood states. These findings confirm that the life satisfaction is expressed in everyday affective experience, but they also show some dissociations between predictors of life satisfaction and of mood, supporting the use of multiple criteria for well-being. We observed different sets of TP predictors for each of mood dimensions, which confirms their uniqueness and provides a further rationale for the three-dimensional model of mood (Matthews et al. 1990).

This findings have several potential applications to studies of well-being, in addition to demonstrating the importance of TP. First, while TP is assessed as an individual trait, scores on the ZTPI show meaningful variation across nations (Zimbardo and Boyd 2008), and cultural variation in TP may contribute to cross-cultural differences in wellbeing and economic growth. (Sircova et al. 2015).

Second, the research reiterates the value of using multiple criteria for SWB, consistent with the psychometric discriminability and differential correlates of its cognitive and affective components (Jovanović 2015; Luhmann et al. 2012). Third, Boyce et al. (2013) point out that typically stable traits may be more malleable than commonly supposed, suggesting individual-level interventions to enhance quality of life. Temporal dynamics are key factors in both anxiety and depressive disorders (Wells and Matthews 2015) and treatments might be designed to support more constructive retrieval, attentional and self-regulative processes as the person reflects on past and future emotion. Indeed, Sword et al. (2015) have developed Time Perspective Therapy on the basis of Zimbardo and Boyd’s (2008) theoretical principles to promote an adaptive, balanced time perspective.
